# Myocardial infarction causes inflammation and leukocyte recruitment at remote sites in the myocardium and in the renal glomerulus

**DOI:** 10.1007/s00011-013-0605-4

**Published:** 2013-03-08

**Authors:** Neil Ruparelia, Janet E. Digby, Andrew Jefferson, Debra J. Medway, Stefan Neubauer, Craig A. Lygate, Robin P. Choudhury

**Affiliations:** 1 Division of Cardiovascular Medicine, Radcliffe Department of Medicine, John Radcliffe Hospital, University of Oxford, Headley Way, Oxford, OX3 9DU UK; 2 Division of Cardiovascular Medicine, Radcliffe Department of Medicine, The Wellcome Trust Centre for Human Genetics, University of Oxford, Roosevelt Drive, Oxford, OX3 7BN UK

**Keywords:** Inflammation, Myocardial infarction, Leukocytes, Remote sites, Renal glomerulus

## Abstract

**Rationale and Objective:**

Acute myocardial infarction (AMI) results in the recruitment of leukocytes to injured myocardium. Additionally, myocardium remote to the infarct zone also becomes inflamed and is associated with adverse left ventricular remodelling. Renal ischaemic syndromes have been associated with remote organ inflammation and impaired function. Here, we tested the hypothesis that AMI results in remote organ (renal) inflammation.

**Methods:**

Mice were subjected to either AMI, sham procedure or no procedure and the inflammatory response in peripheral blood, injured and remote myocardium, and kidneys was studied at 24 h.

**Results:**

AMI resulted in increased circulating neutrophils (*P* < 0.001) and monocytes (*P* < 0.001). mRNA for inflammatory mediators significantly increased in infarcted myocardium and in remote myocardium. VCAM-1 mRNA was increased in both infarcted and remote myocardium. VCAM-1 protein was also increased in the kidneys of AMI mice (*P* < 0.05) and immunofluorescence revealed localisation of VCAM-1 to glomeruli, associated with leukocyte infiltration and increased local inflammatory mRNA expression.

**Conclusions:**

We conclude that in addition to local inflammation, AMI results in remote organ inflammation evidenced by (1) increased expression of mRNA for inflammatory cytokines, (2) marked upregulation of VCAM-1 in renal glomeruli, and (3) the recruitment and infiltration of leukocytes in the kidney.

**Electronic supplementary material:**

The online version of this article (doi:10.1007/s00011-013-0605-4) contains supplementary material, which is available to authorized users.

## Introduction

Acute myocardial infarction (AMI) results in the activation of the acute phase response [1, 2] and mobilisation and recruitment of leukocytes to the site of infarcted myocardium [3, 4]. Furthermore, myocardium that is remote from ischaemic zones has also been associated with the activation of pro-inflammatory pathways and infiltration of leukocytes [5], responses which are increasingly recognised as important in post-infarct left ventricular (LV) remodelling [2]. Renal ischemia syndromes have been associated with remote organ inflammation [6–9] including in the lungs [10] and brain [11, 12]. Substantial myocardial infarction is associated with multiple organ dysfunction, raising the possibility that activation of inflammatory pathways may extend beyond the heart to other critical organs.

Endothelial activation, marked by the expression of specific adhesion molecules, promotes leukocyte recruitment. For instance, upregulation of vascular cell adhesion molecule-1 (VCAM-1) is observed in the contralateral kidney in a mouse model of unilateral kidney ischaemia [13] and in remote myocardium of experimental models of AMI [5], whilst other adhesion molecules (e.g., intercellular adhesion molecule 1 (ICAM-1)) which are constitutively expressed remain unchanged [14]. Infiltrating leukocytes are a source of cytokines, such as IL-6, TNF-α and IL-1, which are involved in both the initial cellular inflammatory response and also subsequent tissue repair and fibrosis after injury [15]. IL1RN, a member of the IL-1 cytokine family, inhibits the activities of IL-1 and has been shown in clinical studies to be associated with higher plasma levels of C reactive protein (CRP) and IL-6 [16]. IL1R2 is a regulator of innate immunity and is the predominant IL-1 binding protein found in monocytes, neutrophils and B cells. It acts as a decoy receptor by sequestering IL-1 (which induces its expression) in the serum and tissues, thus preventing its downstream effects [17]. IL1R2 is significantly upregulated remotely in the lungs (where it is associated with inflammation and impaired function) in a murine renal ischaemia model [6]. IL-1 pathways are of particular interest since IL-1 antagonists (e.g. Anakinra, Canakinumab) are currently being studied in clinical trials [18, 19] in the context of acute myocardial infarction.

The inflammatory response resulting from AMI is systemic, with increased levels of soluble circulating inflammatory markers in humans and experimental models [20], and with the degree of cytokine release positively correlated with adverse outcomes [21]. TNF-α is strongly associated with the occurrence of reperfusion injury after recanalization [22] of the infarct-related artery and increased risk of mortality [23]. TNF-α, IL-6 and IL-1 are significantly elevated in patients with AMI complicated by cardiogenic shock when compared to patients with uncomplicated AMI [24]. IL-1 and IL-6 levels that are elevated in the first 2 days of hospitalization in unstable angina are associated with an increased risk of in-hospital coronary events [25]. Furthermore, cardio-renal syndromes type 1 and type 3, where acute dysfunction in the heart induces decreased kidney function and vice versa, are increasingly recognised but poorly understood [26, 27]. Acute kidney injury (AKI) is common in patients hospitalised with AMI and occurs in approximately one in five patients [28, 29]. Even the development of relatively mild renal impairment in AMI is associated with increased mortality [30–32] and morbidity including development of permanent renal impairment, progression to end-stage renal failure (ESRF) [30], increased length of hospital stay and cost [33].

Whilst the effects of acute kidney ischaemia have been shown to result in a pro-inflammatory cascade and release of cellular and soluble mediators with local [13] and systemic implications [6–12], there are no in vivo animal studies specifically addressing the effects of myocardial ischaemia on the kidneys acutely (i.e. in the first 24 h). Here, we tested the hypothesis that acute myocardial infarction results in remote organ (renal) inflammation, using a mouse model of myocardial infarction to study the inflammatory response in peripheral blood. To do so, we compared injured and remote myocardium and kidneys of mice that received ischaemic injury with those who received sham operation.

## Materials and methods

### Ethics statement

This study was undertaken with the approval of the University of Oxford Clinical Medicine Ethical Review Committee and procedures were conducted in accordance with the UK Home Office Animals (Science Procedures) Act 1986 (HMSO UK).

### Animals

Female C57BL/6 J mice were purchased from Harlan (Blackthorn, United Kingdom) at 16 weeks of age and were housed in ventilated cages with a 12-h light/dark cycle and controlled temperature (20–22 °C), fed normal chow and given water ad libitum. All experiments were conducted on the mice at 24 weeks of age (*n* = 12–15/group). All surgery was performed under isoflurane anaesthesia, and all efforts were made to minimize suffering. Mice were sacrificed by exsanguination under terminal anaesthesia.

### Mouse model of acute myocardial infarction

Following induction of anaesthesia with isoflurane, mice received 0.27 mg of buprenorphine and ventilated with a tidal volume 250 μL and respiratory rate 150/min (Hugo-Sachs MiniVent type 845, Harvard Apparatus, UK). Anaesthesia was maintained using 2 % isoflurane in 100 % oxygen. A left thoracotomy was performed in the fourth intercostal space; the left lung was deflated using a small piece of gauze soaked in saline. The pericardium was then removed and an intra-myocardial ligature placed 1–2 mm below the atrioventricular groove using a 6-0 polyethylene suture (Ethicon, UK). Lungs were then reinflated before the thorax was closed. Sham mice underwent the same protocol with the exception of the ligation of the coronary artery. Baseline (non-operated) mice underwent no operative procedure.

### Murine transthoracic echocardiography

At 24 h following surgery, anaesthesia was induced and maintained with 2 % isoflurane and 98 % oxygen. Development of acute myocardial infarction following surgery was confirmed visually intra-operatively by the absence of blood flow to the anterior myocardial wall and at day 1 following surgery with the presence of a regional wall motion abnormality on transthoracic echocardiography. Transthoracic echocardiography was carried out with a Visualsonics Vevo 2100 ultrasound system (Amsterdam, The Netherlands). Fractional shortening percentage was calculated in both parasternal and short axis views to assess myocardial function/size of infarct using the following formula: end-diastolic diameter − end-systolic diameter/end-diastolic diameter × 100.

### Flow cytometry

Whole blood was obtained following terminal anaesthesia via left ventricular puncture (*n* = 5/group). Red blood cells were lysed with lysis buffer (eBioscience, San Diego, USA). Following washing in fluorescent activated cell sorting (FACS) buffer [phosphate buffered saline (PBS), 1 mM ethylenediaminetetraacetic acid (EDTA) and 1 % bovine serum albumin (BSA)], cells were incubated with a Live/Dead cell viability stain (Invitrogen) for 30 min at room temperature. Cells were then incubated with anti-FcgRII/RIII blocking antibody (BD Pharmingen, Oxford, UK) for 15 min and then incubated with the following antibodies: CD11b-APC, Ly6G-PE (both BD Pharmingen) and CD115-PerCP (eBioscience). Cells were fixed in 1 % PFA and analysed using the flow cytometer (CyAN ADP Flow cytometer, Dako, Ely, UK). Data were then analysed using FlowJo software, version 7.6.3 (Tree Star Inc, Oregon, USA), and compensation and gates were set using single-stain controls, IgG controls and fluorescence minus one.

### RNA extraction and quantitative RT-PCR

Myocardium and kidney tissue were both mechanically homogenised in TRIzol and RNA was extracted with chloroform and ethanol (*n* = 6–8/group) and purified using an RNeasy mini kit (Qiagen, Crawley, UK) as per the manufacturer’s instructions. 1 μg of total RNA was reversed transcribed using a QuantiTect Reverse Transcription kit with oligo dTs and random hexamers as primers. Real-time PCR was carried out with 1 μl of cDNA in a 10 μl reaction mix using Sybr Green Gene Expression assays (Applied Biosystems, Warrington UK) and the primers as outlined in Table 1. Quantification was performed by the 2^−ΔΔCT^ method [34], normalised to the housekeeping gene cyclophilin.

**Table 1:** Primer sequences for quantitative real-time RT-PCR

Target	Sense primer (5′-3′)	Anti-sense primer (5′-3′)
Cyclophilin	GGCCGATGACGAGCCC	TGTCTTTGGAACTTTGTCTGCAA
IL6	CTTCCTACCCCAATTTCCAATG	TTGGATGGTCTTGGTCCTTAGC
TNF-α	AGACCACGTCGTAGCAAACCA	ACAACCCATCGGCTGGCACC
VCAM-1	TCTTACCTGTGCGCTGTGAC	ACAGGTCTCCCATGCACAA
IL1RN	ACAAGGACCAAATATCAAACTAGAAGAA	GCCCAAGAACACACTATGAAGGT
IL1R2	AGTGCAGCAAGACTCTGGTACCTA	AGTTCCACAGACATTTGCTCACA

### Western blot

Protein lysates were obtained following mechanical homogenization of fresh kidney tissue in cell lysis reagent for mammalian tissues (Sigma, UK) (*n* = 6/group). Samples were separated by SDS-PAGE (4–12 % Bis/Tris resolving gel, Invitrogen, Paisley, UK) and transferred to polyvinylidene difluoride (PVDF) membranes using the iBlot^®^ dry blotting system (Invitrogen, Paisley, UK). Immunoblotting was performed using VCAM-1 antibody (R&D Systems, Abingdon, UK). Immunoreactive bands were visualised by chemiluminescence (GE Healthcare Life Sciences, Amersham, UK). Signal intensity was analysed using ImagePro Plus version 6.1 (MediaCybernetics, Silver Spring, MD) and normalised to glyceraldehyde 3-phosphate dehydrogenase (GAPDH).

### Histology

For assessment of myocardial infiltrating leukocytes, haematoxylin and eosin (H&E) staining was performed. At 24 h after myocardial infarction, mice were exsanguinated and immediately perfused with phosphate buffered saline (PBS), and their hearts were harvested and fixed in 4 % paraformaldehyde and embedded in paraffin. Sections (5 μm) were stained with H&E reagents for histological examination. Ten random fields of view (FOV) were obtained at 40 × magnification on light microscopy at mid-cavity level of the left ventricle. The infarct zone was selected anatomically as the anterior wall (as confirmed on transthoracic echocardiography by the presence of a regional wall abnormality) with the remote myocardium selected as the contralateral wall. Infiltrating leukocytes were identified based on their morphological appearance and counted (*n* = 4/group).

### Immunohistochemistry

After the mice were sacrificed as described above, their kidneys were embedded in optimal cutting temperature (OCT) compound. Sections (8 μm) were incubated with a rat anti-mouse CD11b antibody (BD biosciences, UK) and then an anti-rat secondary antibody conjugated to Alexa-fluor 750. Images were obtained using a LSM510 Meta confocal system (Zeiss, UK). Ten random FOV were obtained for each kidney (*n* = 6/group) and positive cells were analysed using ImagePro Plus version 6.1 (MediaCybernetics, Silver Spring, MD). Other sections were incubated with a rat anti-mouse VCAM-1 primary antibody (Santa Cruz Biotechnology Inc., Santa Cruz, CA) alone or in combination with a goat anti-mouse PECAM-1 primary antibody (R&D Systems, Minneapolis, MN, USA). An anti-rat secondary antibody conjugated to Alexa-fluor 750 was used to detect VCAM-1 and an anti-goat secondary antibody conjugated to Alexa-fluor 488 to detect PECAM-1. Images were obtained and analysed as described above. Unstained and secondary antibody-only stained sections served as negative controls and IgG controls were conducted to exclude non-specific binding.

### Evaluating renal function

Plasma was obtained by centrifugation of whole blood. Serum creatinine levels were measured using a mouse creatinine assay kit (*n* = 4/group) (Chrystal Chem, Inc., IL, USA).

### Statistics

Data are expressed as mean and SEM and tested for significance using Student’s *t* test or one-way ANOVA. All statistics were carried out using GraphPad Prism software (La Jolla, CA, USA). A two-tailed *P* value of < 0.05 was considered statistically significant.

## Results

### Acute myocardial infarction

**Fig. 1:**
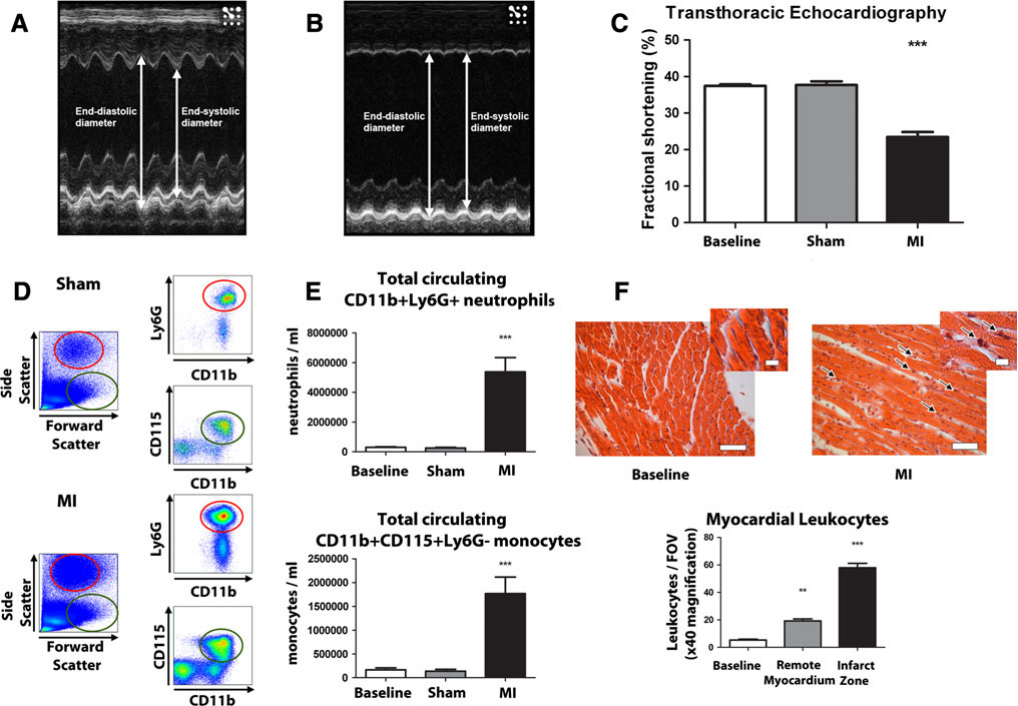
AMI results in impaired LV function, peripheral leukocytosis and infiltration of leukocytes to myocardium. M-mode echocardiographic measurements of left ventricular function through the mid-cavity of a sham-operated mouse (**a**) and an infarcted mouse (**b**) resulting in a significant reduction in left ventricular fractional shortening (**c**) (*P* < 0.001). AMI results in a significant systemic inflammatory response with a systemic leukocytosis (neutrophilia (*red gate*, *P* < 0.001) and monocytosis (*green gate*, *P* < 0.001)) (**d**, **e**). H&E staining of myocardium (× 40 magnification, *scale bar* 50 μm; inset × 100 magnification, *scale bar* 10 μm) reveals significant infiltration of leukocytes to infarcted myocardium in comparison with sham-operated hearts (*black arrows*, **f**) *P* < 0.001

As expected, fractional shortening (FS) was significantly reduced in mice subjected to myocardial infarction, compared to both sham-operated and non-operated animals at day 1 following surgery (*P* < 0.001; Fig. 1a–c). At day 1, MI results in a significant leukocytosis with increases in circulating CD11b + Ly6G + neutrophils and CD11b + CD115 + Ly6G- monocytes (*P* < 0.001) in comparison to non-operated and sham-operated animals (Fig. 1d, e).

### Myocardial infarction results in local inflammation of the ischaemic zone

**Fig. 2:**
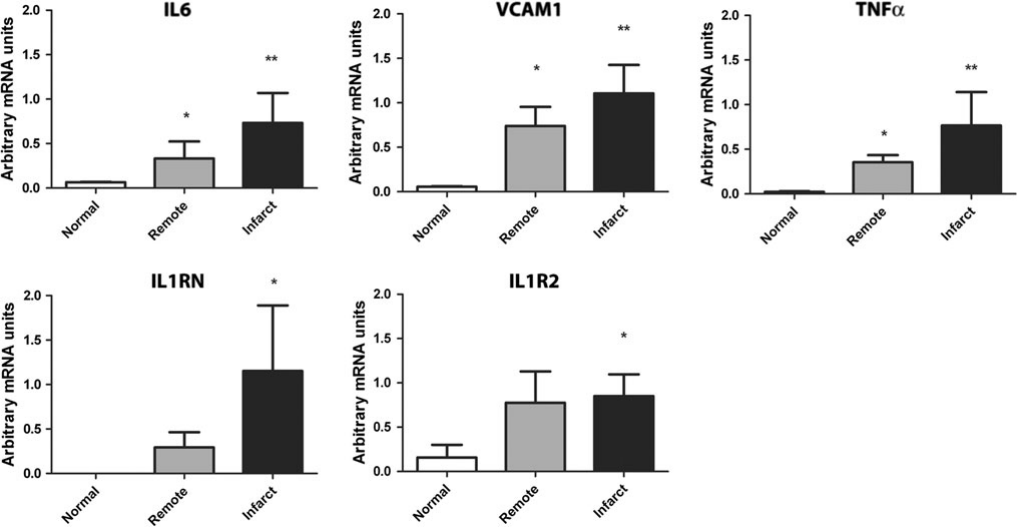
Myocardial mRNA expression of normal myocardium, infarcted myocardium and remote myocardium. In comparison to normal (baseline) mice there were significant increases in IL-6 (*P* < 0.01, *P* < 0.05), VCAM-1 (*P* < 0.01, *P* < 0.05) and TNF-α (*P* < 0.01, *P* < 0.05) in both the remote zones and infarct zones respectively at 24 h. Significant increases in IL1RN (*P* < 0.05) and IL1R2 (*P* < 0.05) in infarct zones in comparison to normal myocardium were also observed. Whilst there was a trend of increase in IL1RN and IL1R2 in remote myocardium, these did not reach statistical significance

H&E staining revealed greatly increased numbers of leukocytes in the myocardial infarct zone, by 10.8 ± 3.24-fold (*n* = 4/group, *P* < 0.001, Fig. 1f). PCR of infarcted myocardium revealed upregulation of inflammatory mRNA expression of IL6 by 11 ± 0.33-fold (P < 0.01), TNF-α by 32.94 ± 2.34-fold (P < 0.01), IL1RN by 5.41 ± 0.24-fold (*P* < 0.05), and IL1R2 by 4.31 ± 0.98-fold (*n* = 8/group, *P* < 0.05). VCAM-1 mRNA expression in myocardium was also increased by 19.62 ± 1.11-fold (*P* < 0.01) (Fig. 2).

### Myocardial infarction results in inflammation of remote myocardium

In addition to investigating inflammation in the infarct zone, we investigated whether there was inflammation in myocardium that was remote from the ischaemically injured areas. H&E staining revealed significantly increased numbers of leukocytes in the remote myocardium by 3.9 ± 1.1-fold (*n* = 4/group, *P* < 0.01) (Fig. 1f) in comparison to baseline myocardium which was associated with upregulation of mRNA for inflammatory mediators. PCR revealed significant increases in mRNA expression of IL-6 by 5.01 ± 0.73-fold (*P* < 0.05), TNF-α by 15.18 ± 4.23-fold (*P* < 0.05) and VCAM-1 by 13.12 ± 2.15-fold (*P* < 0.05) in comparison to baseline (Fig. 2).

### Remote inflammation in the kidney

**Fig. 3:**
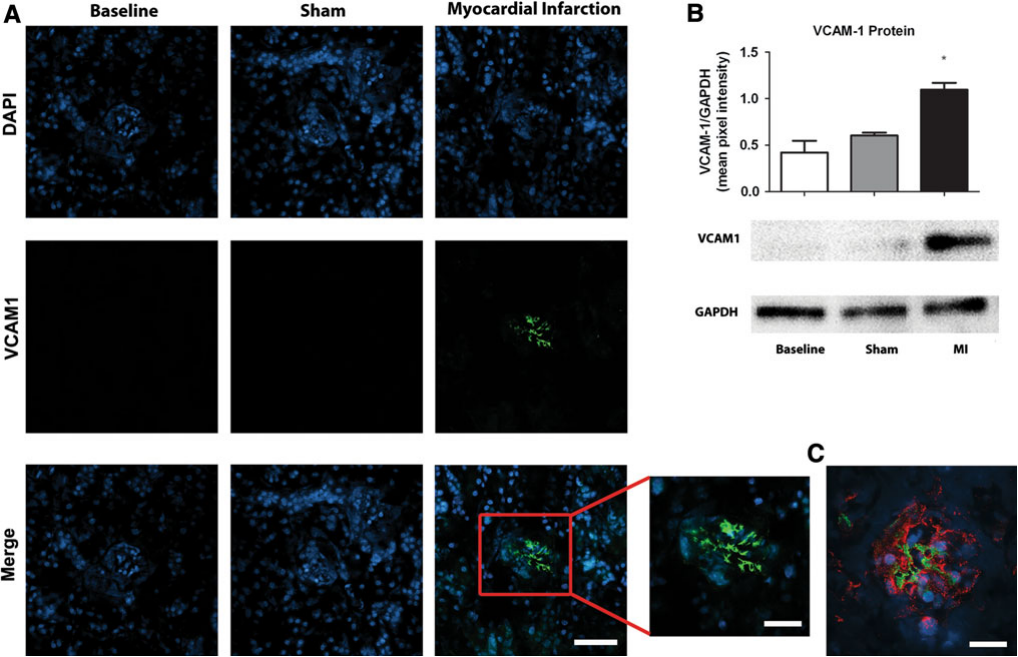
Renal VCAM-1 is upregulated as a result of AMI. Renal VCAM-1 expression (*green*, DAPI *blue*) is significantly upregulated in kidneys of infarcted mice in comparison to baseline and sham-operated animals. The VCAM-1 was found to be exclusively localised to the glomeruli, **a** with the same cells also expressing the endothelial cell marker PECAM-1 (**c**). Renal VCAM-1 protein was significantly increased in comparison to uninfarcted and sham-operated animals (**b**) (*P* < 0.05). × 63 magnification, *scale bar* 50 μm, zoomed inset: *scale bar* 25 μm

Having observed the upregulation of VCAM-1 in both the infarct zone and remote myocardium we proceeded to quantify VCAM-1 mRNA and protein in the kidney. Whilst VCAM-1 mRNA expression was not significantly elevated in the kidney at the time point examined (1.39 ± 0.51-fold; *P* = n.s.) (Fig. 5), VCAM-1 protein was significantly increased in kidneys of infarcted mice (Fig. 3b) in comparison to baseline and sham-operated mice by 1.81 ± 0.14-fold and 2.6 ± 0.27-fold, respectively. Immunofluorescence showed that VCAM-1 was not expressed under basal conditions but was upregulated and clearly localised to glomeruli in mice with myocardial infarction (Fig. 3a) and to the same cells expressing PECAM-1, indicating upregulation by glomerular endothelial cells (Fig. 3c).

### Myocardial Infarction results in increased leukocyte infiltration to remote kidneys

**Fig. 4:**
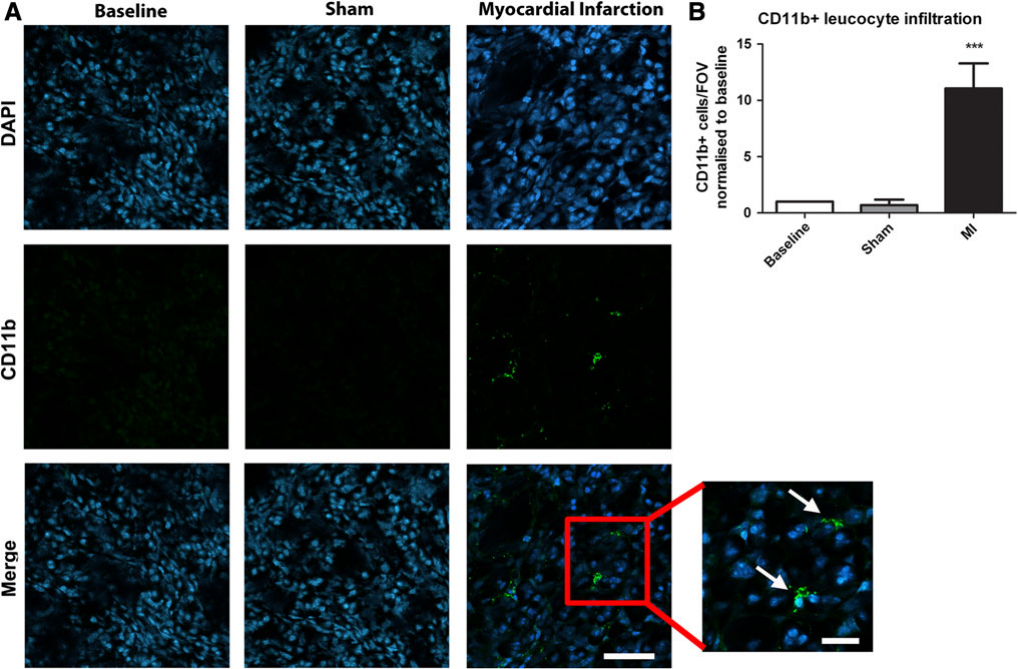
AMI results in significant leukocyte recruitment to remote kidneys. AMI results in a significant increase in infiltrating CD11b + leukocytes (*green*, DAPI *blue*) to remote kidneys in comparison with baseline or sham-operated animals (*white arrows* (inset), *P* < 0.001). × 63 magnification, *scale bar* 50 μm, zoomed inset: *scale bar* 25 μm

In kidneys of mice with acute myocardial infarction we found that there was an increase in numbers of CD11b + leukocytes throughout the kidney by immunohistochemistry (Fig. 4). In contrast, in mice that did not undergo surgery and in sham-operated mice, only occasional CD11b + leukocytes were visualised.

### Myocardial Infarction results in remote kidney inflammation

**Fig. 5:**
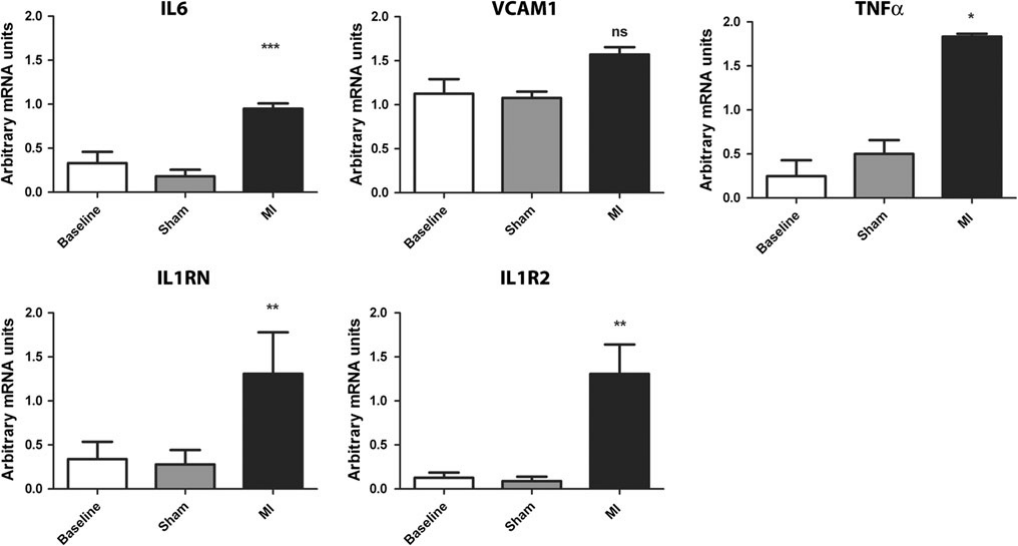
Renal mRNA expression of baseline, sham-operated and infarcted animals. In comparison to baseline and sham-operated mice, IL-6 (*P* < 0.001), TNF-α (*P* < 0.05), IL1RN (*P* < 0.01) and IL1R2 (*P* < 0.01) were significantly increased. At 24 h, VCAM-1 mRNA expression was not found to be significantly different from baseline or sham-operated mice

Having observed increases in both VCAM-1 and leukocyte infiltration in the kidneys, we next sought to investigate the effects of myocardial infarction on mRNA for other inflammatory mediators. Several inflammatory genes were also significantly upregulated in the kidneys of infarcted mice in comparison to baseline and sham-operated animals: IL6 by 2.87 ± 0.13-fold and 5.28 ± 0.07-fold (*P* < 0.001), TNF-α by 7.41 ± 1.8-fold and 3.67 ± 0.15-fold (*P* < 0.01), IL1RN by 3.86 ± 0.19-fold and 4.69 ± 0.28-fold (*P* < 0.01) and IL1R2 by 10.3 ± 0.33-fold and 14.57 ± 4.9-fold (*P* < 0.01) (*n* = 8/group) (Fig. 5).

### Local inflammation in the acute setting has no effect on plasma creatinine

Acute myocardial infarction, did not affect plasma creatinine (baseline: 8.25 ± 0.3 μmol/l vs. MI: 10.33 ± 2.0 μmol/l, *P* = n.s.) despite evidence of renal inflammation.

## Discussion

There is an increasing appreciation that systemic inflammatory pathways are activated in a variety of medical conditions (e.g. AMI, severe sepsis, trauma, renal ischaemia) and that these pathways play an important role in the development of remote organ inflammation leading to adverse outcomes. In experimental models of acute kidney injury (AKI), remote organ (heart [9, 35], lung [36], liver [8] and brain [12]) inflammation has been described, resulting in dysfunction. The acute effects of AMI (first 24 h) on remote organ inflammation have not previously been investigated. Here, using a mouse model we investigate the acute effects of AMI on inflammation remotely in the kidney. The present study demonstrates that: (1) AMI induces a local inflammatory response, characterised by infiltration of leukocytes and increased expression of mRNA for inflammatory cytokines; (2) cytokines are upregulated in myocardium that is remote from the site of ischaemic injury; (3) AMI is associated with systemic inflammation, reflected in increased peripheral blood neutrophils and monocytes; (4) AMI is also associated with remote organ inflammation evidenced by (a) increased expression of mRNA for inflammatory cytokines, (b) marked upregulation of VCAM-1 in renal glomeruli and (c) by the recruitment and infiltration of leukocytes throughout the kidney.

Infiltrating leukocytes to areas of acute myocardial inflammation and post-MI myocardium are a source of inflammatory cytokines (e.g. IL-6, IL-1 and TNF-α) and contribute to local inflammation, remodelling and fibrosis of the myocardium [37, 38]. It has recently been observed that leukocytes also infiltrate *remote* myocardium post-MI, as a consequence of increased expression of adhesion molecules and inflammatory cytokines, and may contribute to adverse left ventricular dilatation, fibrosis and impaired function [5].

De novo renal impairment or AKI in the setting of AMI affects 20 % of hospitalised patients [28, 29, 32]. The degree of impaired renal function is associated with increased short- and long-term mortality [30, 31, 39], and in this setting even mild transient renal dysfunction is an independent risk factor for long-term survival [28].

Upregulation of IL-6, IL-1 and TNF-α in the kidney may contribute to acute renal dysfunction and also subsequent fibrosis and chronic impairment [40, 41]. The expression of VCAM-1 exclusively in the glomeruli of the kidneys supports recent observations of glomerular VCAM-1 expression as a result of TNF-α stimulation in keeping with acute inflammation [42].

We found that this was associated with a significant increase in leukocyte infiltration which suggests that endothelial activation at remote sites may play a role in initiating or exacerbating renal inflammation and its sequelae with the same glomerular cells expressing PECAM-1 (Fig. 3c). However, glomerular mesangial cells may also play a role in VCAM-1 expression and monocyte infiltration [43].

There are a number of possible mechanisms that could explain these findings. Soluble mediators such as inflammatory cytokines (IL-6, TNF-α) released from ischaemic myocardium may act directly at remote areas. Studies have shown upregulation of mRNA expression of cytokines and resultant phenotypic evidence of injury associated with dysfunction [44] with blockade of these cytokines attenuating remote inflammation and injury in various ischaemic models [9, 45, 46]. Increased inflammatory cytokines result in increased apoptosis in cardiomyocytes [9] and, additionally, an increase in oxidative stress in hepatocytes as a consequence of AKI [7]. Remote organ inflammation may also result from cellular mediators both as part of the innate and adaptive immune response. The innate immune response may be activated by the activation of toll-like receptors (TLRs) and release of reactive oxygen species or by the activation of the complement pathway as a result of ischaemic tissue which in turn would lead to the release of cytokines and endothelial activation with trafficking of leukocytes [47]. Adaptive immunity may also be activated with T cells having been shown to play a central role in renal ischaemic models [48] in both the acute inflammatory response [49] and also long-term renal dysfunction [50]. In addition to cellular mediators, there also may be other key modulators, e.g., lectin-like oxidized low-density lipoprotein receptor-1 (LOX-1), which can serve as a pro-inflammatory adhesion molecule [51] and whose expression and activation can lead to the generation of reactive oxygen species [52]. Abrogation of LOX-1 in a mouse model has been shown to reduce inflammation, recruitment of leukocytes and the development of fibrosis in kidneys following AMI [41].

It is clear that the mechanisms governing remote organ inflammation are complex and may all contribute to different degrees in addition to contributions from other factors such as microparticles [53] and neurogenic pathways. Most of our current understanding results from renal ischaemic models with the effects of AMI on remote organs and the underlying mechanisms for this remaining poorly characterised.

A major current limitation in clinical practice is the absence of a biomarker that is sensitive enough to detect small changes in renal function. Routinely used measurements such as serum creatinine or blood urea nitrogen start to rise only after a significant reduction in renal function, though novel biomarkers such as kidney injury molecule-1 (Kim-1) and lipocalin-2 (Lcn2) may hold some promise for the future [54]. Renal expression of VCAM-1 could potentially be used as a biomarker for renal inflammation post-MI to identify patients at risk of AKI in this scenario [13].

In view of these findings, there may be a role of emerging drugs in this patient group with drugs such as IL-1 antagonists (e.g. Anakinra, Canakinumab). Indeed, interleukin-1 blockade in a mouse model of AMI [55] and in a small clinical pilot study has been found to be safe in AMI and favourably affected left ventricular remodelling [18]. Thus these drugs may have a role in the management of AKI in AMI, resulting in improvements in short-term AKI and also in the development of long-term renal fibrosis, ultimately improving long-term mortality and morbidity.

There are limitations to this study. A correlation between the magnitude of myocardial inflammation and the extent of remote organ inflammation could be beneficial in identifying patients at higher risk of developing remote organ injury at the bedside. However, due to small numbers and the marked variability of the magnitude of inflammation, we had insufficient numbers for correlation analysis. Additionally, we were unable to demonstrate functional significance of remote kidney inflammation: creatinine (as used here) is an insensitive marker for glomerular filtration rate (GFR), however there are currently no better measures available. Finally, poor cardiac function and therefore renal hypoperfusion may account for some of our findings and the sham surgery group does not adequately control for this variable. However, in similar experimental models, the blood pressure reduction following AMI has only been found to be modest [41], and in other ischaemic models (e.g. renal ischaemia) remote organ inflammation has been found in the absence of any change in cardiac function [7–10], suggesting that blood pressure would not be the only mechanism to explain our findings.

In summary, this study demonstrates remote kidney inflammation at 24 h following AMI, characterised by endothelial activation, recruitment of infiltrating leukocytes and the release of local inflammatory mediators. On the basis of these findings, further work is merited to establish the time course of renal injury following AMI and the effect of remote kidney inflammation on function.

## Electronic supplementary material

Below is the link to the electronic supplementary material.

ESM1 (AVI 5039 kb)
